# An Internet of Things Example: Classrooms Access Control over Near Field Communication

**DOI:** 10.3390/s140406998

**Published:** 2014-04-21

**Authors:** Daniel Palma, Juan Enrique Agudo, Héctor Sánchez, Miguel Macías Macías

**Affiliations:** University Center of Merida, University of Extremadura, Sta., Teresa de Jornet, 38, Mérida 06800, Spain; E-Mails: dpalmage@alumnos.unex.es (D.P.); sasah@unex.es (H.S.); miguel@capi.unex.es (M.M.M.)

**Keywords:** Internet of Things, NFC, Arduino, sensors, smart environment, classroom access control

## Abstract

The Internet of Things is one of the ideas that has become increasingly relevant in recent years. It involves connecting things to the Internet in order to retrieve information from them at any time and from anywhere. In the Internet of Things, sensor networks that exchange information wirelessly via Wi-Fi, Bluetooth, Zigbee or RF are common. In this sense, our paper presents a way in which each classroom control is accessed through Near Field Communication (NFC) and the information is shared via radio frequency. These data are published on the Web and could easily be used for building applications from the data collected. As a result, our application collects information from the classroom to create a control classroom tool that displays access to and the status of all the classrooms graphically and also connects this data with social networks.

## 1. Introduction

The effective management of classrooms, halls, offices, and public spaces in any institution or building is often a difficult problem. There are many rooms of different types within a building; therefore, recording the activities undertaken in them in real-time is usually intricate. Thus the smart room concept described in this paper tries to solve this problem by using sensing solutions, intelligent environments or decision making environments [1,2]. Furthermore, there are many classrooms or labs in universities with a variety of equipment and purposes that are used for several subjects during an academic year. Though these classes are assigned at the beginning of the year, this can usually be changed throughout the course. When any change occurs, it is difficult to communicate within the institution and the wider university community.

In the light of the aforementioned context, there is an idea that has gained strength in recent years: the Internet of Things. The Internet of Things connects all the objects of everyday life such as washing machines, shoes, televisions, *etc.* to the web, allowing us to access information in real time and from anywhere about this object [3]. There are many applications that are taking this technology whose major purpose is to facilitate the use and management of aspects of our daily life. One of the most extended uses is remote sensing such as fire control, which uses a sensor network throughout a forest to monitor the temperature in order to prevent potential fires [4].

In addition, we have other related Internet of Things technology, such as Near Field Communication (NFC) technology [5], a short range wireless technology that can be used to obtain information through identification labels. In some universities in Spain (including the University of Extremadura), the identification cards for both teachers and students possess this technology, which can be used to develop applications.

Related to the Internet of Things, there is free platform hardware closely linked to this technology, Arduino [6], a project whose goal is to provide simplicity in creating electronic applications. This platform has a good reputation as an electronic prototype development board because of its ease of use and the affordability of its components. Many of the applications developed with Arduino use Web 2.0 to display or collect information. There are a lot of developers in this community, sharing their knowledge through blogs and social networks and allowing anyone to use the applications they develop or the information they have managed. This information exchange is crucial as it allows the creation of projects based on this information to create or enhance new applications.

As already stated, the main objective of this work is to provide each classroom with a system to collect information about its real time use: establish a user identification method in each room that can be accessed from the centre, using items that the university already has, take advantage of the opportunities that the Internet of Things provides by creating an application to record the use of the classrooms. This article is organized as follows: in Section 2 the technologies involved are introduced. In Section 3 the proposed architecture for classroom access control is specified. Section 4 concludes with the work completed as well as the proposals for future work.

## 2. Technologies at Work

In this section each of the technological aspects mentioned in this paper (the Internet of Things, Arduino, Web 2.0 and NFC) is analyzed in turn.

### 2.1. The Internet of Things

In the beginning, the Internet was only designed for communication in which computers could access websites, download content or communicate with other users. However, technologies evolve creating more powerful devices, faster and with more capabilities. Advances in electronics technology are also creating smaller devices with low power consumption which means that large networks of sensors can be created, with the ability to obtain information, process it and act accordingly. Here it is how the idea of the Internet of Things arises [3]. This term was coined in 1999 by Kevin Ashton, cofounder of Auto-ID Center at the Massachusetts Institute of Technology (MIT) [7] bearing in mind the concept of “ubiquitous computing” [8,9]. Under this term, computers and technologies are around users without noticing their presence, being able to cooperate and adapt their behaviors to the environment and enabling users to interact with technology without interfering with their everyday life. In this sense, the concept of computer as hardware device is diluted to integrate connected devices around and in cooperation with users' daily life. This research group was created with the idea of developing another emerging and innovative technology, RFID codes, and the belief that thoughts ideas and knowledge information are important and things and objects are crucial. However, the information we have today depends heavily on the data generated by users in their interactions with objects. Throughout the years, a lot of information (images, files, data, music, *etc.*) has been kept which, thanks to the possibilities offered by the Internet, can be accessed from anywhere. The problem lies in the amount of time, dedication and precision required for the collection of all this information.

When the term “the Internet of Things” appeared it was done under the premise that if there were devices with the capacity to know everything about such objects or things. From all the data previously gathered we would be able to track and control everything and to know when things need to change [10,11]. If all our objects (shoes, appliances, books, *etc.*) have small identifying devices, we may collect information from them constantly from any place. At the company level this would allow managers to have absolute control of products and stock. On a personal level, there would be improvements in many areas of our life, for example the end of thefts.

The characteristics of any device to be integrated into the Internet of Things are [3]:

- Collect and transmit data: the devices are in environments in which information can be collected and either sent to another device or directly to the Internet.
- Operate action-based devices: devices can be programmed to act according to any condition.
- Receive information: they can receive information from the network.
- Support communication: the device belongs to a network of devices that can communicate with each other through other nodes in the same network.

One of the things that make it special and different is that the Internet of Things allows objects to communicate directly or indirectly to Internet. Furthermore, this information does not have to be used only by the user, but by other people for applications or studies. In terms of architecture, it is “event driven”, built from the bottom up (based on the context of processes and operations in real time) and takes into account any additional level. This implies that the Internet of Things would not be subject to certain rules and regulations, but able to handle exceptions and evolve based on the context of the event, but could also be adapted to common standards, if necessary.

### 2.2. Arduino

The open hardware platform Arduino is closely related to the previous section [12]. Arduino is a platform that seeks simplicity when creating applications by using a combination of software and hardware; it is based on a board with a single microcontroller input/output pin for communication and control of physical objects and the environment. This kind of device has been developed to connect all kinds of objects and its functionality directly relates it to the Internet of Things.

This project was launched in 2005 in order to create control devices for projects that were easy to use and inexpensive to acquire. Initially the project sought to teach students electronics quickly and easily. Massimo Banzi, one of its creators, was asked to perform this task. The product consisted of cheap parts that users can easily find to create their own boards. However, the key decision was to ensure that Arduino was “plug-and-play” that someone could connect the board to one computer and use it immediately. The Arduino philosophy was that if someone wanted to learn electronics they could do it from day one without having to start from the beginning. Soon the project gained popularity for its simplicity as much as for its low price in basic models ($30). Due to its success, different versions with various capabilities have been developed (UNO, Mega, Leonardo, Mini, Due, Yún, *etc.*) [13]. Arduino UNO has been chosen because of its compatibility with the other models since it is the last version of the original model. Also it offers enough capacity and functionalities to this project and in case more portability or connectivity issues are needed, we can expand it with extra shields or easily move to another board, for example Arduino Mini (smallest one) or Arduino Yún (Wifi connection). Arduino Ethernet is also used to provide Internet connectivity but it is simply an Arduino UNO that has Ethernet connector instead of USB connector.

Basic Arduino boards use Atmel AVR microcontrollers that have 8-bit modified Harvard RISC architecture. These microcontrollers are very simple and low cost which makes them ideal for this type of project. Arduino UNO is a microprocessor board with Atmega328 with 32 KB memory, 2 KB of RAM and 1 KB EEPROM. It also has 14 digital input/output pins (6 can be used as PWM outputs), 6 analog inputs, a ceramic resonator 16 MHz, a USB connection, a power jack, an ICSP header, and a button to restart [6].

**Figure 1.**
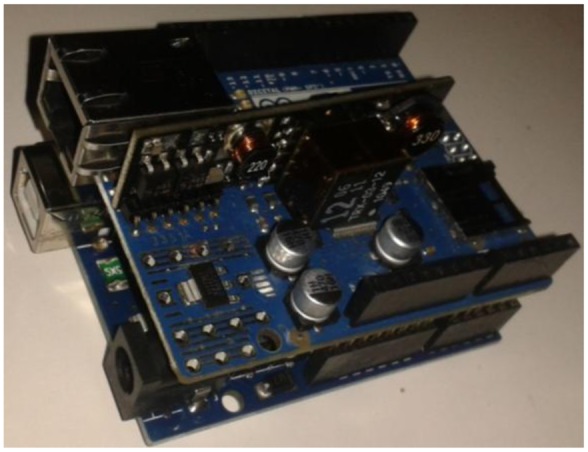
Arduino UNO and Ethernet shield.

Arduino shields are another of the key factors for Arduino success (Figure 1). These boards allow easy assembly with Arduino models. Shields have the same pin at the same location as the Arduino models, so when they are used the pins don't have to be wired. This also enables its use by those without a great knowledge of electronics. With these shields we can also extend its communication capabilities via Ethernet, Wifi, GPRS or Xbee. Currently there are 317 shields by 125 different manufacturers in the Arduino Shield List [14].

### 2.3. Web 2.0 and Mashups

The term Web 2.0, attributed to Tim O'Reilly [15], arises from the concept that the Web should be an “architecture of participation”, a platform for information that allows innovation by independent developers. The idea was also that it was free of use, to allow more people to participate, combined with the concept of mobility as many users from different places can be involved.

Web 2.0 refers to those websites that facilitate information sharing, interoperability, user-centered design and collaboration with the World Wide Web. This term includes communities, services and Web applications, social networking, video hosting services, wikis, blogs, mashups and folksonomie. Some of these associated services include:

- Blogs: they are personal spaces where authors can write articles and news in chronological order and a collaborative space where readers participate.
- Wikis: a collective website construction with a specific topic, in which users are free to add, remove or edit content.
- Social Networking: Web sites where each user has their own page which allows them to post content and communicate with other users.
- Resource sharing environments: they are places to store all kinds of data in order to share and view them from anywhere (documents, videos, photos, *etc.*).
- Folksonomies: is the work of tagging Web content. The value of this action lies in that people can use their own vocabulary to explicitly add value to the content they are consuming.

In Web 2.0 there are two technologies widely used to exchange data between applications, JSON or XML:

- XML (eXtensible Markup Language) is a software technology based on information derived from SGML language and developed by the World Wide Web Consortium. The files are XML documents whose information is organized in a tree which is used for exchanging structured information on different platforms and could be validated with XML-Schemas. In fact XML is a meta-language that could be used to define our own communication language.
- JSON, JavaScript Object Notation, is a lightweight alternative to XML format for sending or receiving data. JSON belongs to a subset of the object literal notation of JavaScript. JSON is a collection consisting of pairs of name/value. Since these structures are in any programming language, we can say that the exchange of data using JSON is independent of the programming language used. This has been one of the keys to its growing popularity if simplicity is sought.

The term “mashups” [16] refers to a page or Web application that takes data from one or more sources to present it in a totally different way to the original site. Integrating is quick and easy by using open source and shared data to create a distinct service from that initially provided. The architecture of a mashup consists of three parts: the content provider, that is the source of the data which may be available via API or different Web protocols such as RSS, REST or Web Services, the Site Mashup, which is the application that is created from the foreign data and, finally the client Web browser, which is the user interface of the mashup.

Google Maps [17] is one of the clearest examples of mashup, a web application server that offers images of maps and photographs from all over the world via satellite, from every street as well as routes between different locations. Google Maps relies on its development in JavaScript and XML. Google Maps allow free use but can also be customized. Changing the color of the maps, using your own pictures to display a particular place as well as the addition of information through tables or symbols are for example the way you can customize your map. Initially, the user uses the Google map server for viewing. Once downloaded, changes don't go through the server (which is obvious), so Google uses AJAX technology to make these changes without going through the server to create an interactive application. These applications run in a user's browser and communicate in the background. AJAX is an asynchronous technology, meaning that additional data is requested from the server and loaded in the background without interfering with the display and behavior of the page.

### 2.4. Near Field Communication

Near field communication (NFC) is a wireless short-range and high frequency communication technology that allows the exchange of data between devices. This technology is based on electromagnetic fields [5] that usually communicate by using an identification card with a reader. The standard governing the use of electronic identification cards and smart cards is ISO 14443 (RFID), which is managed by the International Organization for Standardization (ISO) and the International Electrotechnical Commission (IEC) and FeliCa. NFC works in the field of 13.56 MHz and shares the characteristics of the ISO 14443.

NFC Smart cards are not used only in card format. This technology can be integrated into cards, keychains, bracelets and mobiles. In addition, Smartphones with this technology can also be used to exchange information or to read passive RFID tags. Its transfer rate can reach 424 kbit/s. This is somewhat limited, so its use is usually intended for other purposes. NFC is not used to transfer large amounts of data; their main aim is instant communication.

The maximum range of NFC is 20 cm. Although this may seem to be a disadvantage, it does not have to be, as this is what leads to improved security. Also NFC teams are able to send and receive information simultaneously. Because NFC devices must be in close proximity to each other, usually not more than a few centimeters, they have become a popular choice for secure communication between consumer devices such as Smartphones. Therefore peer-to-peer is a defining feature of NFC. An NFC device can act as both a reader and a label. This is one of the features for their use in mobile payment. In our case, MiFare cards are selected since are extensively used in access control for office buildings, payment systems for public transport, as well as other applications [18].

## 3. Architecture Design for Classrooms Access Control

The operation of this project is governed by the functionality of the Internet of Things [3]. Our classrooms registration system is based on a network of connected sensors that collect information that is then uploaded to the cloud so that any application can use this information when necessary. This scheme has two distinct parts which include:

- The “thing”, which contains in our case is the classroom and its access.
- The cloud, which are Internet applications where the data is stored.

**Figure 2.**
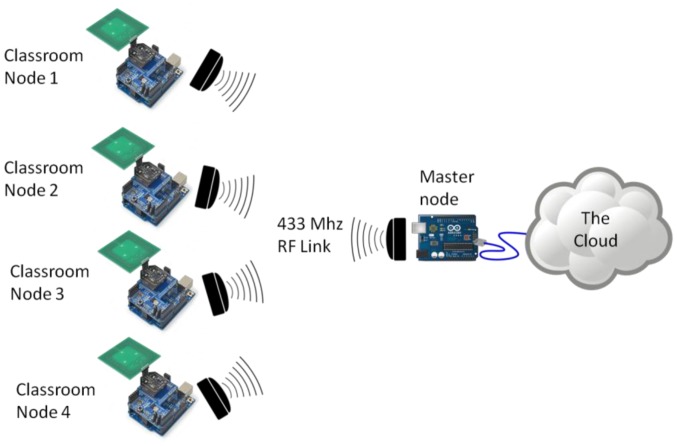
System communication scheme.

The overall process is described in Figure 2, where each class collects the data of interest through NFC, which is sent through the RF and then stored in the cloud so that the information can be accessed by the application.

**Figure 3.**
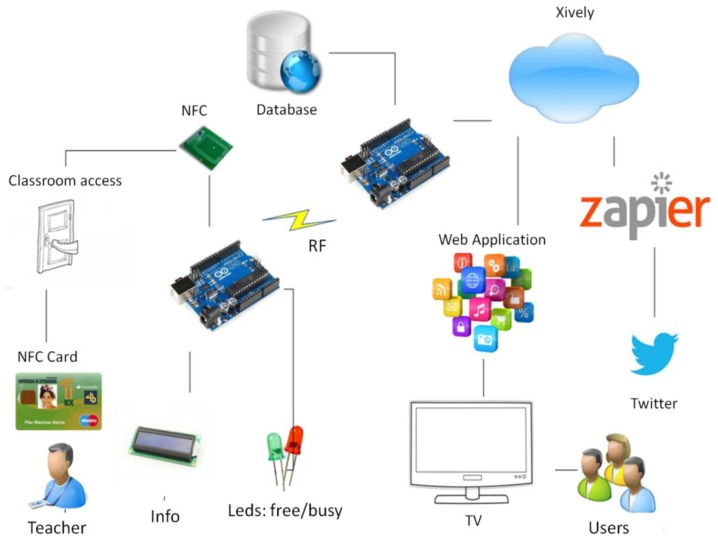
Overall system.

In more detail, the system would start working when trying to access a classroom (Figure 3), a process where an ID card would be used for this purpose. The user uses an NFC card reader connected to the Arduino device that shows a message on the LCD screen and one of the LEDs lights up, showing that the classroom is in use (red LED).

Immediately afterwards, the Arduino device connects to another Arduino (Master) by radio to identify the user accessing the classroom system. The system will use this ID to connect to the database and check whether it is a teacher and retrieve their name.

The next step is sending the data to the cloud, transferring the name of the teacher and the classroom to the server platform Xively [19] to be conveniently stored.

From the data stored in Xively, two applications are created. On the one hand, Google Maps is used for retrieving data from Xively to create a map of the university showing the classroom information. This application can be accessed from any computer and the contents can be displayed on the university information screens (TV panels).On the other hand, an application could send messages with information from each classroom by taking advantage of the social network Twitter (tweets). To help us accomplish this task, the tool Zapier is used [20].

### 3.1. Hardware

**Figure 4.**
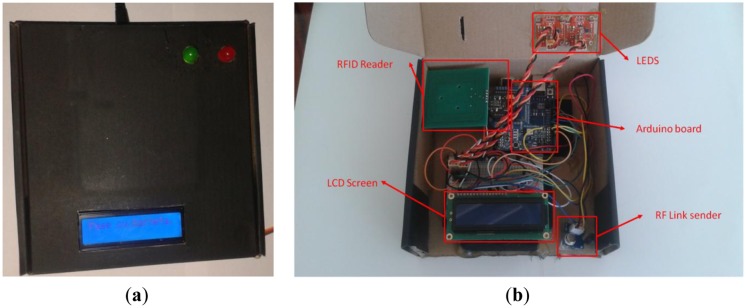
Classroom node hardware prototype: (**a**) outside (**b**) inside.

In this section the hardware used in the two types of nodes is described. Classroom nodes that read the card sent by RF are composed of (Figure 4):

- Arduino UNO.
- NFC module for Arduino and communication shield from cooking-hacks [21].
- LCD screen,
- Two LEDs (Red and Green),
- Radio Frequency module (433 MHZ).

**Figure 5.**
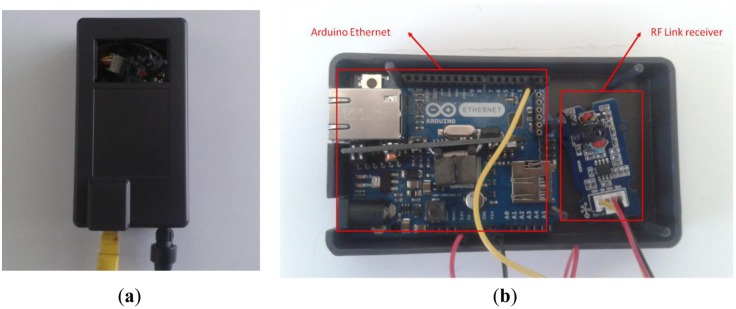
Master node hardware prototype: (**a**) outside (**b**) inside.

On the other hand, the master node is responsible for receiving the RF information and sends it to the cloud through Ethernet so it includes (Figure 5):

- Arduino Ethernet.
- Radio Frequency module (433 MHZ).

### 3.2. Reading Data (NFC)

The collection of data through user interaction with an NFC reader is the initial offstage in the system. The description of the reader and how it works is presented below. Our NFC reader consists of an Arduino Uno, a Xbee shield with an NFC reader, a radio frequency module, an LCD display and two LEDs. Before attempting to access the classroom, it could be either free or occupied. If it is free it will be possible to complete the identification process and access the classroom.

**Figure 6.**
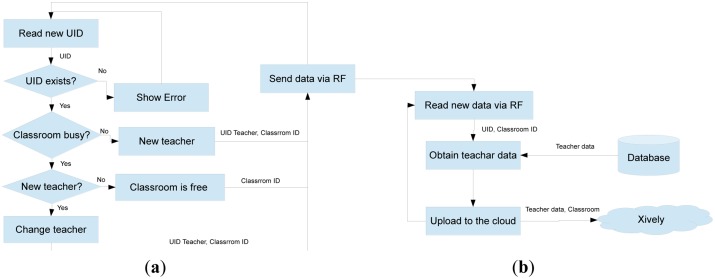
System flowchart diagram (**a**) reading cards (**b**) uploading data to cloud.

Figure 6a (see above) shows the reading process of cards. First the reader will read and identify two facts: ID and user name. In the NFC, and MiFare RFID technologies, the ID card is called UID. The UID is the first four bytes to be found in block 0 of sector 0. The other data should have been already saved. To record this information, keep in mind that each block is composed of sectors of 16 bytes. The write and read position is the address memory 4.

Once the player has collected this information, it displays a message on a screen with information on the user's name and ID in hexadecimal. If the state of the classroom is busy this is displayed on the LCD screen by turning on the red light and sending the information read by radio. To send data, the reader uses the NFC RF module on the computer using Arduino Uno pin 6 to send the data. Once the data has been sent, the reader saves the state of the class, occupied or empty. When a user wants to leave the classroom and thus change the status, they must swipe their card through the reader, which then follows the process described above and confirms the output of this with a new message and by lighting the green LED.

At this point a third use case is considered. It may be that a user leaves the classroom forgetting to swipe him/her card, making the status remains busy. In this case, a new user being aware of this condition, passes their card and the reader identifies the new ID and checks with the new user to ensure that this is a change. From here, the system will work just as described above.

Security solutions in RFID or RF communications have not been considered because of the information sent (card ID and classroom ID) and its use in this way is not critical. But in case that the system needs improving its security the RF communication could be encrypted [22] via software or choosing more secure transmission solutions as Zigbee. Regarding the software, the RMF12B library [23] could be used so that we just need a very small size algorithm for Arduino devices. Likewise, it provides encryption with XXTEA algorithm that operates with 64-bit blocks and uses 128-bit keys which is a strong alternative talking about small solutions [24]. In the Zigbee case, it includes the encryption as an option since it is based on a 128-bit AES algorithm incorporating the strong security elements of the IEEE 802.15.4 standard [25]. This option needs hardware modification, including an Xbee ZB device [26] and a Bee socket [27] in both nodes Zigbee module will communicate via serial with Arduino.

### 3.3. Processing Data

**Figure 7.**
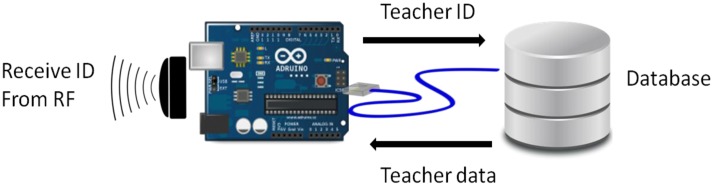
Processing data.

The master node is the destination of the data that the NFC reader has obtained and sent by radio frequency. The master consists of an Arduino Ethernet and RF module with a receiving antenna (Figure 7).

Initially, the receiver will connect to the network by using its Ethernet connection and prepare the necessary pin (pin 2) for receiving data via radio frequency. Once done, it will remain in standby until it receives the data.

Once the receiver has collected data by the NFC reader, it must perform processing to complete this information. Keep in mind that when reading NFC, it is transmitting the identification code for each user and the classroom they want to access, while finding the name of the user involves consulting the database hosted on the server. To place a PHP service in the same server through an HTTP request from our Arduino, the user name and related information will be returned when the ID is received from the reader. This information will be required to store data in the cloud, as discussed in the following section. In order to get a good performance in the master node, the data is uploaded to the cloud immediately and no information is saved on it (Figure 6(b)).

### 3.4. Data in the Cloud

Since the major goal of this project is mainly focused on the access to the classroom by teachers, the response from the server database to the http request can only contain the teacher's name. Thus, if the person were not registered in the database, it would imply that such user is not a teacher, or an authorized person, and access would not be consequently registered.

Once the data of an access are read, it is sent to our server to map the classrooms so that the results are displayed and are visible to everyone. This data is only sent to Xively when the state changes instead of updating it periodically.

The explanation of how the Xively server works is briefly described below. Xively provides a working environment in which you can create a new project for each application. Each project consists of a number of variables called “channels” that store the values of our applications. In our case, a channel for each classroom that we will use is created.

**Figure 8.**
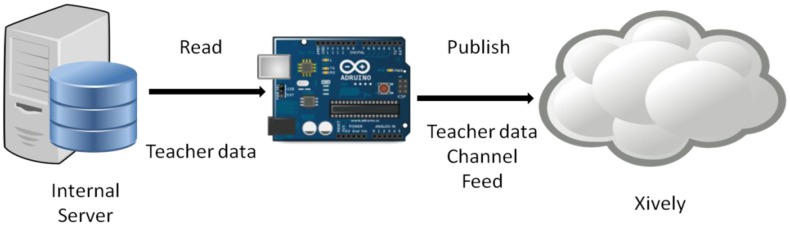
Putting data in the cloud.

The program created in Arduino, takes the name of the teacher as a variable and uses the PUT function of Xively and the appropriate parameters (key to our account on the server and classroom ID) to update the server data (Figure 8). To make use of these functions, first the Xively page is checked to obtain a password to use their API, then the project is identified as “feed” and the Xively library to Arduino is downloaded.

### 3.5. Using the Data: Google Maps App

**Figure 9.**
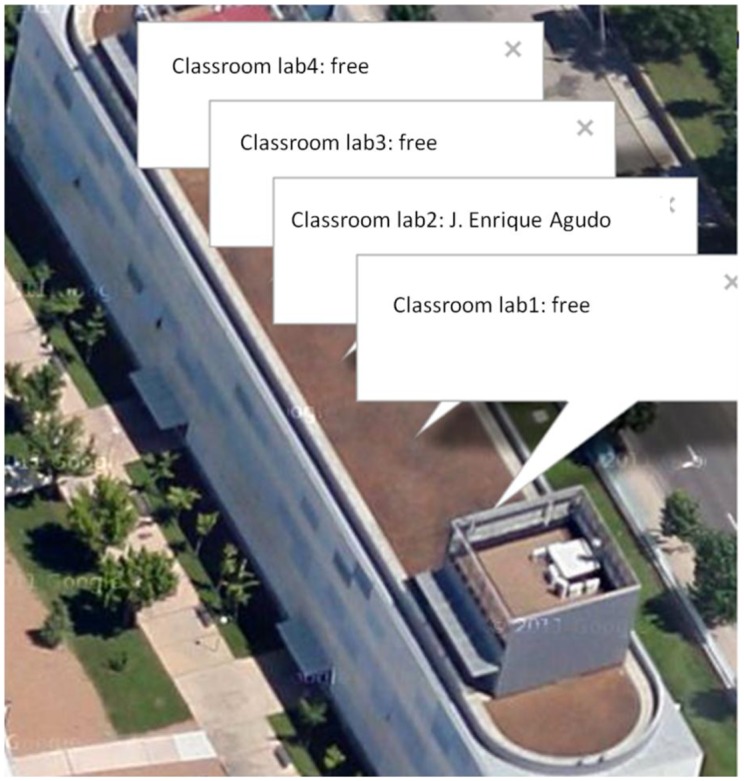
Data in the map.

In this part, the data are already stored on the server, which means that they can be accessed from anywhere from any application. An application that makes use of that data is created. The application will consist of a combination of Google Maps with data stored in Xively. The application shows a map of the University Center of Merida in which information boxes for each classroom appear (Figure 9). If a teacher is in a classroom, teacher's name will appear on the corresponding box.

To implement this application, three major elements are used: Xively API, Google Maps API and JSON technology. In order to use API Xively, it will be necessary to establish the same values as in the previous section: the project feed and the API key. As for the Google API, you can access Google Maps as developers to get the key and to work with the maps.

The JSON language is also used to combine both applications. In this case the XivelyJS library which allows us to use this system is used. Therefore the application will retrieve data from Xively to build information boxes in Google Maps. The application will be accessible from any browser and does not need to be refreshed as any change in the status of the classrooms will be reflected in the application instantly.

### 3.6. Reusing Data: Social Networks

This section consists of creating an application that allows us to publish data stored in Xively in a social network by using the tool Zapier. This tool is easy to use and has been chosen for its high interoperability. At present you can connect over 250 Web apps. One only needs to decide [20] which application best suits his/her need.

Zapier is a service that allows us to combine several Web applications. With Zapier tasks can be performed automatically because it allows certain actions in the application or website to cause various tasks to be performed in a second application. Zapier defines this type of action as a zap: “When a new thing comes to A, B does something else”. Zapier allows great customization of tasks by defining each field within an application to be set: the text that is sent, regularity, parameters, *etc.*

Besides customization, Zapier allows a large number of shares and a large number of tools to be used. Each of these services allows an action in an application (e.g., receiving an email in Gmail) to be reflected in another, separate application (for example by adding a reminder in Evernote).

**Figure 10.**
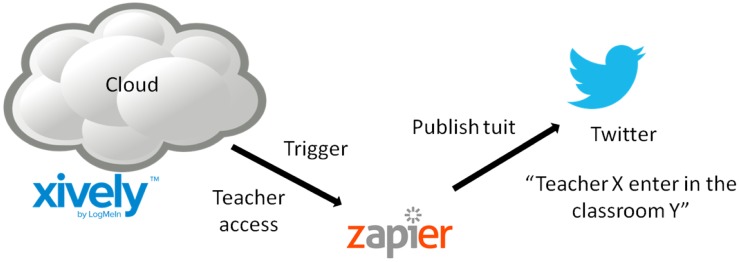
How Zapier sends tweets.

One of these services, called Web hook is being used. Web hook involves taking data from Xively and posting it elsewhere-in this case in the social network Twitter (Figure 10). Whenever a change occurs in any of the channels (classrooms), Zapier automatically sends a tweet to the account indicated showing the change and state.

## 4. Conclusions and Future Work

A tool and a device have been developed to manage classrooms in real time. To do this, a transmitter/receiver access control for each classroom has been created, data are uploaded to the cloud and a Web application to view the data has been built.

Arduino hardware has provided enormous simplicity in the development of the prototype. The different modules and shields are successfully integrated. The programming of the device was not complicated as the Arduino developer community is large and there are many tutorials that can be accessed via the net.

The most important part was the development of the Web application. Using Google API for their maps, combined with the data hosted on the Xively server, has been the key to this project. This work has shown an example of integrating different technologies by using the basic principles of the Internet of Things. The result is that a thing, the classroom, registers information in the cloud via a sensor network created with Arduino components. Different applications that make use of data by integrating them into an application that uses Google Maps to produce information published on Twitter has been developed. This not only shows the possibilities of the Internet of Things but also scalability and reuse of data that can be generated. Also NFC, RF, Arduino, Xively, Google Maps and Zapier technologies are combined in the same project with a successfull result that tests the power of the Internet of Things for managing and sharing data.

Therefore, it should be noted that the possibilities exist not only in controlling access to classrooms but in the use of information stored in the cloud to create new applications. This system offers the solution to a problem but also lays the foundation for a series of future projects that could lead to additional improvements as follows.

More roles for access to the classroom could be integrated into the project. In the project only the role of teacher is taken into account. However, one could add the roles of non-teaching staff and students, such as allowing access to staff without a record of activity in the Web application or allowing students access at set times.

The system functionality could be increased by placing a relay and automatic locks allowing the door of the classroom to open when a card with authorized access is swiped. In addition, by placing motion sensors, or sensors that measure temperature, sound or light, to detect use, would remove the need for the teacher to swipe a card when entering the classroom. In the application server and Xively, the subject and schedule of teacher could be added, as well as the equipment needed and the student roster.

Finally, the data shared in social networks could be used for additional purposes by combining it with other applications such as updating the school Google Calendar.
